# A Wireless Sensor Network for Vineyard Monitoring That Uses Image Processing

**DOI:** 10.3390/s110606165

**Published:** 2011-06-07

**Authors:** Jaime Lloret, Ignacio Bosch, Sandra Sendra, Arturo Serrano

**Affiliations:** 1 Integrated Management Coastal Research Institute, Universidad Politécnica de Valencia, C/Paranimf, n° 1, 46730, Grao de Gandia, Spain; E-Mail: sansenco@posgrado.upv.es; 2 Institute of Telecommunications and Multimedia Applications (iTEAM), Universidad Politécnica de Valencia, Camino Vera n/n, 46022, Valencia, Spain; E-Mails: igbosroi@dcom.upv.es (I.B.); arsercar@upvnet.upv.es (A.S.)

**Keywords:** Wireless Sensor Network, image sensor, image processing, vineyard monitoring

## Abstract

The first step to detect when a vineyard has any type of deficiency, pest or disease is to observe its stems, its grapes and/or its leaves. To place a sensor in each leaf of every vineyard is obviously not feasible in terms of cost and deployment. We should thus look for new methods to detect these symptoms precisely and economically. In this paper, we present a wireless sensor network where each sensor node takes images from the field and internally uses image processing techniques to detect any unusual status in the leaves. This symptom could be caused by a deficiency, pest, disease or other harmful agent. When it is detected, the sensor node sends a message to a sink node through the wireless sensor network in order to notify the problem to the farmer. The wireless sensor uses the IEEE 802.11 a/b/g/n standard, which allows connections from large distances in open air. This paper describes the wireless sensor network design, the wireless sensor deployment, how the node processes the images in order to monitor the vineyard, and the sensor network traffic obtained from a test bed performed in a flat vineyard in Spain. Although the system is not able to distinguish between deficiency, pest, disease or other harmful agents, a symptoms image database and a neuronal network could be added in order learn from the experience and provide an accurate problem diagnosis.

## 1. Introduction

The vine is one of the oldest crops. It is believed that the *Vitis vinifera* cultivation has its beginnings in an area between the Black Sea and Caspian Sea. The first evidence of vine cultivation in Spain dates from 520 BC and grape growing and winemaking spread throughout the peninsula during the Roman time.

Spain is the first country in the world in land devoted to wine production, featuring a total of approximately 1.1 million hectares. It is followed by other countries like France and Italy, according to the balance of the global wine situation made by the International Organization of Vine and Wine (OIV) in 2009 [1]. When we talk about wine production, Spain generates approximately 13.1% of the total world wine. With these indicators, it is not difficult to conclude that this type of cultivation is very important for some of the European countries and they need to keep it in good conditions in order to ensure, in each harvest, the good performance and quality of the grapes and wine [2].

There are some European regulations that cite the economic relevance of diseases in grapevine, regarding some pathogens. For example, the directive 68/193/CEE of the European Council [3] cites some virus diseases: Grapevine Fanleaf Virus (GFLV), Arabis mosaic virus (ArMV), Grapevine leafroll-associated virus 1 (GLRV-1), Grapevine leafroll-associated virus 3 (GLRV-3) and Grapevine fleck virus (GFkV). This text has been revised several times. The last version is the directive of the European Parliament and of the Council COM (2010)359 [4]. There are very few regulations and directives about vine deficiencies. The oldest law we have found is from 1901 [5]. It is an Australian Act to consolidate the Acts relating to a certain vine disease and to vegetation diseases. New Zealand’s Legislation has some laws about pests, deficiencies, and diseases such as the one presented in [6] about Bisosecurity, that includes grape vines.

Nonetheless, it is known that some diseases, such as diamine putrescine [7] or leafroll [8], can cause visible damage to the grape (hindering their sales and production), as well as to the vine. They can cause the reduction of grape production, with consequent economic losses. But these losses could be caused by other reasons. An example of a non-parasitic apoplexy was recorded in Lanzarote during the harvest of 1994, where economic losses of up to 60% occurred in some regions [9]. It is important to remember that because the plants do not assimilate the nutrient treatments immediately, there can also be losses in the subsequent restoration years of the vineyards after the infection. This phenomenon occurs because most diseases and deficiencies often affect the most young branches and shoots, which hinders the proper growth of the plant [10].

Generally, a deficiency or excess of some nutrient, the presence of a virus, pest or a parasite on a vine, produces some physical change in the plant, creating anomalous pigmentation, especially in the leaves. For this reason, foliar analysis is quite important [11]. We can distinguish different types of harmful agents in the vine. These are the following [12]:

- Physiological damage or disorders in vine that are not caused by pests and diseases: lack or excess of irrigation, extreme conditions due to meteorological factors, natural aging, deficiencies or excesses of nutrients in the vine, bad soil, irrigation with saline water, damage to roots by tilling, plant damages caused by poorly performed improper pruning.
- Fungus: Most of them attack leafs, grapes and stems. They can even grow well in both wet and dry regions. Some well known fungi are powdery mildew, downy mildew, and bunch rot.
- Vine pests and insects: These problems can be evaluated as they develop. Insects feed on buds, leaves, and grapes. The most damaging are those that feed on grapes because the resulting rot can spread throughout the entire cluster. Some of these are the following: *Tetranychus urticae*, *Meloidogyne*, *Heterodera*, *Ditylenchus*, *Lobesia botrana*, *Colomerus viti*, *Eriophyes vitis*, *Phylloxera vastatrix*, *Haltica ampelphaga*, *Byctiscus betulae* or *Sparganothis pilleriana*, among others.
- Vine diseases generated by viruses and bacteria: virus and virus-like diseases and other infectious diseases of grapevines are induced by intracellular pathogens of various natures. For example, grapevine degeneration, grapevine decline, leafroll complex, rugose wood complex, yellow mottle, line pattern, yellow dwarf, stun, Ajinashika, Fleck, Roditis leaf discoloration, yellow speckle, among others.

There are several ways and methods to diagnose and find out what harmful agent or nutritional deficiency is affecting the vine. These are the following [13]:

- Visual inspection of the crop to check for deficiencies signs: only critical deficiencies are noted, but the damage symptoms observed sometimes can be unreliable.
- Soil analysis: qualifying the soil nutrient levels and other characteristics.
- Analysis of the plant tissue: measurements of nutrient levels in plant tissues. It can detect weaknesses that could not be found in the soil tests.
- Bioassays: these are methods to diagnose nutrient deficiencies which combine the techniques of tissue analysis and testing in pots.
- Field Tests: this test is the oldest and most reliable method to diagnose nutrient deficiencies, but it is an expensive procedure.

These methods are used as a first step in vine exploration, because the field tests are quite expensive and difficult to manage. Besides, usually, it is not possible to perform them on the plant, but rather only in a laboratory.

Over the years, there have been notable technological advances in agriculture. They may be classified according to their nature. We find biochemical products, such as pesticides and fertilizers, farm machinery, such as tractors or irrigation timed systems, and the ground nutrients, among others. From the mechanical and the information technology application perspective, the technological improvements should not disturb the environment. Some examples of information technology applied to the agriculture, rural, and forest areas are applications based on the detection of natural disasters, monitoring and control of agriculture, ecosystems and geophysical measurement systems, flood detection, precision agriculture, biological complexity of environmental mapping, and detection of forest fires [14,15]. Moreover, it is easy to find some deployed sensor systems for monitoring all meteorological parameters of the environment such as temperature, humidity, wind, *etc.* [16] or distributed monitoring systems based on Wireless Sensor Network (WSN) technology [17]. This technology cannot be used to obtain new agricultural products, but rather to improve existing techniques and working conditions of the people in charge of the fields, and to improve disease diagnosis in order to ensure the final product quality. Frequently, these technological advances require a high initial financial contribution, but over the years, this is outweighed by the benefits obtained from the field [18].

In this paper, we propose a WSN that uses an image processing system in each wireless node in order to distinguish if a vine has bad leaves or not. These bad leaves could be caused by a deficiency, pest, disease or another harmful agent. When the wireless sensor detects any unusual status in the vine leaves, it generates an alarm that will be routed to the sink node in order to warn the farmer. The benefits of this deployment are huge and the farmer will recoup the investments of this implementation very fast (the time will depend on where the vineyard is planted, the probability of having deficiencies, pests or diseases, and depending, on how far away the farmer lives and on how many time he/she usually spends observing the vineyard looking for any deficiency, pest or disease). The main contributions of this paper are the following:

- We provide a detailed design of the WSN where we discuss the radio coverage distance and the field of view of the cameras used to detect the deficiency, pest, disease or other harmful agents.
- We also present an innovative way to estimate the number of nodes needed to sense an area.
- While other works describe the design of new sensor devices, we, on the other hand, use a cost effective sensor based on existing IP routers that can be found readily in the market, and we adapt them for our purpose.
- The image processing technique explained in this paper lets us distinguish between the land and the good leaves from the bad leaves in order to detect any symptom caused by a deficiency, pest, disease or other harmful agent.

The rest of the paper is structured as follows. Section 2 describes some related works that shows the state of the art in this research field. The wireless sensor network design, the wireless sensor node deployment and its procedure are explained in Section 3. Section 4 defines bad leaves and explains which type of bad leaves could be detected by our system. Section 5 shows the image processing method for vineyard monitoring. Sensor network traffic measurements are shown in Section 6. Finally, Section 7 draws the conclusions and proposes future works.

## 2. Related Works

A basic machine vision system consists of a camera, a computer equipped with an image acquisition card, and a lighting system. In addition, adequate software should be considered in order to convey the correct signals to electronic equipment in order to successfully perform the acquisition operation, the storage and the image processing process [19]. There are very few technical works about image processing and pattern recognition in precision agriculture. In this section we discuss some of these related works.

In [20], Baidyk *et al.* present two systems for image recognition. They show the structure and the recognition algorithms and the limited receptive area (LIRA) neural classifier. Then, they show several applications for their proposal. The first application is an image recognition system based on adaptive control system for micromechanics where the LIRA neural classifier is proposed for texture recognition of mechanically treated metal surfaces. Based on this first application, and taking into account that agriculture tends to over-use pesticides to kill insects and pests, the authors propose the use of a web-camera based computer vision system in order to automate the task of recognition of larvae. The system is able of recognize the difference between the textures corresponding to the larvae and the real world background. Thus, if the system locates the position of the insects and larvae, the use of pesticides will be local and chemical products consumption will be lower. The proposed system consists of a series of neural classifiers, held together and organized into layers. Each classifier decides based on the captured image, whether the searched parameter is present or not. Based on this decision, this node is labeled as ON or OFF and transmits its result to the next layer. This new layer has a smaller number of classifiers and collects the responses from the previous layer. The answers are transmitted to an upper layer and each layer has a top most comprehensive filters, which will define if the generated image is part of an insect or not. Once the image is formed, it is processed and validated by different combinations of horizontal, vertical and diagonal distortion of the image. Because the recognition of larvae and worms of different sizes, shapes, colors and positions is not a trivial task, the system needs an initial training stage based on the display of different images. With this pre-processing technique, the authors train the system to be able to recognize and extract the larvae of any image with different backgrounds.

Sometimes it is difficult to use machine vision to distinguish the weeds from the main crop in real time, due to the large processing capacity and resources needed for this purpose. Artificial neural networks (ANNs) have evolved and, actually, solve some of these difficulties by interpreting images so quick and effectively. A work based on the same type of system than in the previous work is presented by Yang *et al.* in [21]. The aim of this study is to develop a back-propagation ANN model that could distinguish young corn plants from weeds. In order to perform the various tests, the authors use a series of cameras, which are adjusted to obtain images with sufficient clarity. Each image is preprocessed in bitmap format with the Image Processing Toolbox v2.0 for MATLAB v5.0 and is converted to indexed images based on a red-green-blue (RGB) color system. Each pixel of an image is classified in one category, represented by an integer in the range from 0 (black) to 255 (white). Finally, each assigned color index number serves as an ANN input. After data pass through different layers of the neural network nodes, the system obtains as an output a value of 0 or 1. This value depends on whether the system has been considered that the image has been taken of a useful plant or a weed. The same tests are performed for different types of neural networks (multiple outputs). The authors remark that although it may take approximately 20 hours to properly train the network, if this training is done correctly, the system needs only a second to process each image. This processing and image recognition system could be used in conjunction with an herbicide spraying system in order to improve efficiency and reduce the amount of applied herbicide.

Another work related to the detection of weeds in agriculture is presented by Yang *et al.* in [22]. In this case, the authors present the initial stages of developing a system to capture, and an image processing technique to detect weeds. Using a fuzzy logic system, the authors are able to create a weed map, in order to let the system take decisions on the exact location and the amount of herbicide that can be applied to the field. In order to perform various tests, the authors used a commercial digital camera and a personal computer. The image colors are compared with the intensities of RGB color system. Depending on the color intensity values, they obtain a binary matrix (obtained through the fuzzy logic), which provides information on the green zone of weed cover, and the distribution of weeds. The authors were able to perform such functions with common cameras without the need to use complex devices. In addition, their system is able to control the amount of herbicide that is dispersed into the field, and to reduce the pollution soil and water, generating significant cost savings.

In [23], Sena *et al.* used machine vision for detecting a type of worm (*Spodoptera frugiperda*) that affects maize plantings. This control is generally only performed by pesticides. The authors show the flow diagram and the evaluation results of their algorithm for the identification of maize plants damaged by a worm using color digital images. The proposed algorithm has two stages. In the first stage, the images are taken and converted to gray scale images. Then, they are processed to create binary images using an iterative algorithm. During this process, the leaves are segmented and divided into pixels. In the second stage, the images are divided into blocks and those blocks, which leaf surface is exceeding 5%, are selected. On each selected block, any anomaly is identified and is marked as damaged or undamaged by using the number of objects found in each block. The authors conclude their work performing an efficiency assessment of their algorithm. The studied insect has eight stages of maturation and the efficiency of the proposed algorithm is higher in the first two phases, obtaining 94.72% in the percentage of right answers (considering as affected blocks, those who have at least 10 objects).

Cruvinel *et al.* present in [24] an automatic method based on image processing for measuring the drop size distribution of agricultural sprinklers. In this paper, their authors seek to determine the exact size of the sprinkler drops, because if they are too small, the droplets could be subject to wind drift and could distort the pattern of irrigation application, but if the drops are too big, they acquire more kinetic energy and could lead problems concerning soil erosion, aggregated breakdown, surface sealing, and infiltration. To achieve this goal, the authors present a technique based on image processing, using the properties of Fourier analysis and correlation in the frequency domain. This direct measurement on the sprinkler drops allows studying the effects of pressure and nozzle size on the distribution of water. With this system, farmers would be able to regulate the size of the drops, to adjust the amount of water to rural needs, and take into account factors such as wind.

Macedo-Cruz *et al.* presented an assessment method to classify the land covered with oat crops, and to quantify planting density affected (and not affected) by very low temperatures (frost) in [25]. They use a combination of three thresholding strategies (the Otsu method, the isodata algorithm, and the fuzzy thresholding method) in order to quantifying the damage. They state that this merger gives better results than taking each of them separately. The classification strategy used involves three main procedures: an automatic thresholding, the fusion of thresholds and classification using the merged value, the validation of the classifier by computing the fuzzy error matrix to measure the accuracy. Finally, they show the accuracy of the classifier.

Moreover, the use of cameras can provide numerous benefits over traditional homogeneous sensor networks. An example is provided by Kulkarni *et al.* in [26]. They propose a camera sensor network for object detection, recognition and tracking. Their main contributions are the novel mechanisms introduced for low-power low-latency detection, low-latency wakeups, efficient recognition and tracking. But, their proposal use light image processing techniques to detect the targets, thus it would be a weak system to be used for detecting any type of deficiency, pest, disease or other harmful agents.

Finally, Liu *et al.* investigate the coverage of camera sensor networks for target detection [27]. They propose a localization-oriented sensing model based on the perspective projection of the camera sensors. Then, they analyze how the probability of the localization-oriented coverage changes with the number of sensors and the parameters of the proposed model.

In [28], Istin *et al.* propose a method for real-time distributed image acquisition through wireless networks for traffic monitoring. They also present a collaborative online algorithm, which identifies an optimized set of cameras that must be turned on to cover the field of view loss.

On one hand, we see that the first works presented in this section are only focused on image processing, without taking care in depth how should be the devices used for image detection, how they should be placed to record images and how many of them should be in the field to cover an area. Moreover, all of them develop the work without giving details about the movement of the camera (if is fixed or mobile) or if it could change its direction of view. The second ones are only focused on target detection and tracking, but they do care much about the image processing. On the other hand, none of the previous works show the sensor network design, the wireless sensor deployment, or how the node processes the images for vineyard monitoring. Moreover, the distributed wireless sensor network proposed in this work permits low bandwidth consumption, allowing higher scalability than when all video streams are transmitted through the network, in order to cover larger cultivated areas.

## 3. Wireless Sensor Network and Wireless Sensor Node Deployment

**Figure 1.**
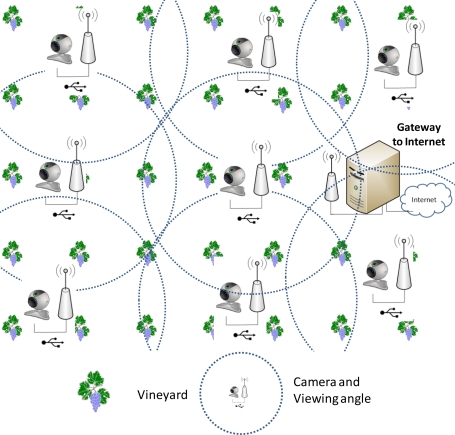
Vineyard monitored by cameras.

The proposed wireless sensor network is based on a set of nodes capable of capturing images, processing them internally in each node and producing an output response based on its decision (if it has found a bad leaf in the field or not). This design is a distributed system where each node makes its own decisions, thus avoiding sending too many messages through the network to a central server in order to make those decisions. Based on our previous studies, we know that each camera, when using a MPEG-4 codec compressed video over HTTP protocol, will require a traffic flow bandwidth of 800 Kbps for a video resolution of 320 × 240 at 25 fps. It implies a maximum of 30 cameras in an IEEE 802.11g network [29], which is not scalable or useful for large fields. This pushed us to make a distributed design process when image processing is needed in the WSN, thus avoiding the limitation of the number of cameras because of the bandwidth requirements. Thus, on a vineyard, we deployed a WSN where the wireless sensor nodes should be strategically placed in order to maximize the sensed area to be covered by the nodes while minimizing the number of nodes to be placed in the field (that is maximizing the wireless radio coverage). Figure 1 shows the designed WSN topology. In this work we will make our design and deployment over flat fields or over smooth hills (which usually happens in almost all vineyards in Spain), but in case of uneven or steep fields, sensor nodes should be placed at the top of the hills or the mountains.

The use of IEEE 802.11 technology requires more power consumption than when using other technologies, such as Bluetooth or ZigBee. But we used it nevertheless in order to cover larger areas because the sensors could be quite far. It allows us to have lower deployment costs because fewer devices have to be deployed. However, we should take into account the energy consumption, so we considered the recommendations published for WLANs by us in [30]. In order to select the best routing WSN protocol for our purposes, we took into account the work presented in [31]. Thus, we selected Optimized Link State Routing (OLSR) protocol as the routing protocol because it is the one that has the best performance for our case. In our design we use a single sink node which is placed in the vineyard house.

Although our initial design is based on the aforementioned statements, we can make use of group-based protocols in order to cover bigger areas [32].

### 3.1. Analytical Network Model for Node Placement

One of the first issues to take into account is the placement of the wireless sensor node. The position of each sensor is determined by two different coverage areas. The fist one is the radio coverage area, which allows the sensors to communicate. The second one is the sensing coverage area. Usually, the radio coverage area of a sensor node is quite different from the sensing area. Moreover, generally, there is no relationship between them. Both types of coverage areas could be affected by the field where the WSN is deployed, but just one of them could be affected. The factors that affect the sensing area are different from the factors that affect the radio coverage area. The goal is to have the physical space within the sensing range of at least one wireless sensor. The number of wireless sensors deployed is usually higher than optimum required for the physical space due to the lack of precise sensor placement.

In a rural and agricultural zone, the radio coverage area for a wireless sensor mainly depends on the type of antenna, used by the wireless sensor and its gain, its height with respect to the top of the vineyard plants and the humidity of the location. In our model we take placement of the wireless sensor at the top of a post of 6 meters height (vineyard plants are not usually more than 2–2.5 meters high), so we will assume that the vineyard plants do not affect to the radio coverage because of the difference in height. In order to estimate the radio coverage we use the power balance formula [given by Equation (1)]. This equation states that the received signal power, in dBm, is equal to the transmitted power plus the transmitter and receiver gain, minus the basic loss and minus other losses produced by other issues (such as vegetation or humidity) [33], but in this case we will only take into account the humidity. The value of humidity losses can be obtained from reference [34]:

(1) $$P_{rx}(dBm)=P_{tx}(dBm)+G_{tx}(dB)+G_{rx}(dB)-10\cdot n\cdot log\;d-L_{humidity}(dB)$$

Here *n* is the attenuation variation index. *n* = 2 for air medium and *d* is the distance between the transmitter and the receiver. So, solving Equation (1) in *d*, we obtain the coverage distance in Equation (2):

(2) $$d=10\frac{P_{tx}+G_{tx}+G_{rx}-L_{humidity}-P_{rx}}{20}$$

In order to estimate the distance between devices we fix some parameters. The theoretical transmitted power is −40.2 dBm for an IEEE 802.11g WLAN device at 1 meter (*P_tx_*) and at 2.44 GHz (mean value in the 2.4 GHz WLAN range). We fixed to −80 dBm the threshold power in order to have enough quality of signal (*P_rx_*). So, the received power must be greater than or equal to this value, otherwise it is not in the radio coverage area. We will consider omnidirectional antennas for both the transmitter and the receiver both with the same *Gain*. Moreover, this study has been done in Spain, which has two main hydrometric areas: the H area and the K area [35], so losses due to rain, in the worst case, have a value of 0,026 dB for two kilometers. Equation 3 shows the formula needed to design the radio coverage of each wireless sensor:

(3) $$d=10\frac{39.77+2\cdot Gain}{20}$$

**Figure 2.**
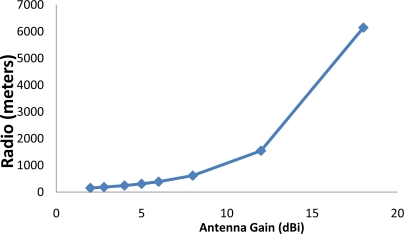
Radio coverage for several typical antenna gains.

Figure 2 shows the radio coverage for several typical 2.4 GHz antenna gains.

The sensing coverage area of a node is also called sensing field (or region) of the node. The characteristics of sensing equipment are completely different and rely on the application requirements. The sensing region of an isotropic sensor in the ideal environment is circular (or elliptical). But a directional sensor has a finite angle of view and thus cannot sense the whole circular area, so its sensing region would be a sector in a two-dimensional plane or a 3D zone.

The most commonly used model for sensing coverage is the disk model, which assumes that the sensing region for a sensor is a circular region centered on it [27]. Any point placed in the sensing radius of the sensor could be sensed. So, any stem, grape or leaf will be covered if is closer than the maximal sensing distance (*d_max_*). When the camera only covers a region (not the whole circle), the target must be located inside the angle of the rotatory movement of the camera. Based on the perspective projection model of a camera sensor, we propose a partial circle model. The area inside a partial circle bounded by a radius *r* and an arc α is provided by Equation (4):

(4) $$Area=\frac{1}{2}d_{max^{2}}\cdot (\pi \cdot \frac{\alpha }{180}-sin\alpha )$$

**Figure 3.**
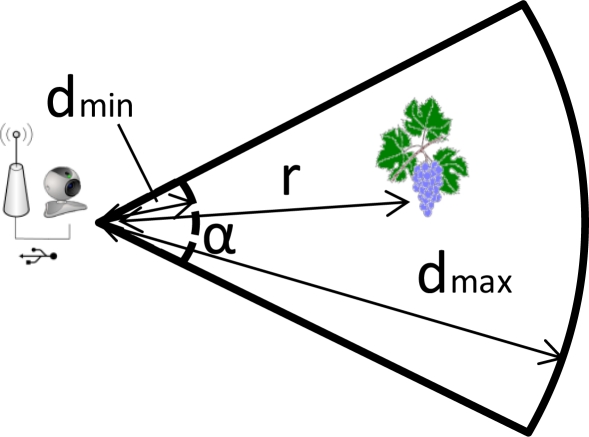
Sensing coverage of a wireless sensor camera.

Here r is the circle radius and α is the angle (in degrees). We also define a minimal sensing distance because the focal length of the camera could be very small, so d_min_ will be the minimal distance of effective imaging. Figure 3 shows the partial circle area covered by a wireless sensor camera. *r* is the distance between the camera and the target.

The field of view (FoV) of a camera (if the image system is rectilinear) depends on the collimator focal length and the target size (assuming the sensed image includes the whole target). It can be estimated by Equation (5):

(4) $$FoV=2\cdot \frac{D}{d}arctan(\frac{d}{2\cdot f_{c}})$$

where *D* is the dimension of the full image, *d* is the dimension of the target and *f_c_* is the focal length of collimator.

Let us assume that all nodes have same radio coverage radio (thus the same radio coverage area) and the same sensing area, but the radio coverage area and the sensing areas are different. Moreover, we assume that all nodes have the same computation capabilities.

Let a network of wireless sensor nodes be *G* = (*V*, *E*), here *V* is the set of wireless sensors and *E* is the set of connections between them. Let us suppose *n* = |*V*| (the number of sensors of *V*) to cover a field, which we refer to as the sensing field. The sensing coverage area of a wireless sensor is denoted by *S* while the radio coverage area is denoted by *C*. The sensing area is different to the radio coverage area (*S* ≠ *C*). For simplicity, we assume a sensing area of a node *i* being a disk centered at sensor *i* with radius *s*. Likewise, a sensor *i* covers a target *q* if and only if target *q* is in the sensing disk of *i*. That is, if *d_min_* ≤ *d(Lc, Lq)* ≤ *d_max_*, here *d(Lc, Lt)* is the Euclidean distance between *Lc* (camera location) and *Lq* (target location), *d_max_* the maximal sensing distance and *d_min_* the minimal sensing distance. It is desired a non-overlapping sensing areas in order to maximize the performance of the network. Two sensors can communicate directly with each other if and only if each sensor is inside the radio coverage area of the other. That is, if *d*(*Lc_1_*, *Lc_2_*) *≤ C*, here *d*(*Lc_1_*, *Lc_2_*) is the Euclidean distance between the camera 1 and the camera 2 and *C* is the radio coverage radius of the wireless sensors (we have assumed that all wireless sensors have the same radio coverage radio). A node that can directly send messages to its neighbors, we call it 1-hop communication.

The position of the wireless sensors on the network coverage area (also called as Region Of Interest, ROI), should be those ones whose coverage percentage is maximized and coverage holes is minimized. There are three main strategies for this:

- Visual forced algorithm [36]: the sensors and the objects to be sensed in the ROI make the placement of sensors away from the objects and also from each other, so that their sensing areas are not overlapping and a full coverage is achieved.
- Grid based sensor networks divide the ROI into square grids and the sensors are placed at the center of each square [37]. Generally, the bigger the grid size with respect to the sensing range the higher accuracy sensing the ROI or the higher probability to object tracking.
- Computational geometry approach is frequently used in WSN coverage optimization. The most commonly used computational ones are the Voronoi diagram and Delaunay triangulation [38].

In our case, the vineyards are usually placed in a flat field where there are not high drops. Moreover, both the radio coverage area and the sensing area are circular areas. So the easiest deployment system for the sensing coverage is the grid-based method and this was applied in our case.

The sensing region is usually divided into two sub-regions: the uncovered region (whose points cannot be sensed) and the covered region (whose points can be sensed). In our deployment we try to have an uncovered region as close to zero as possible.

**Figure 4.**
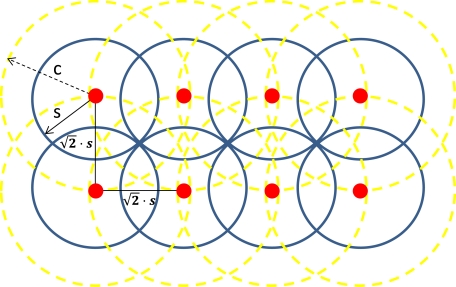
Sensing coverage of a wireless sensor camera.

In order to deploy the wireless sensor cameras we will assume that the radio coverage radius is equal or higher than two times the sensing coverage radius (that is, using previous nomenclature, *c* ≥ 2·*s*). This will allow us to have full coverage of the ROI while maintaining radio connectivity between wireless sensors. Figure 4 shows eight wireless sensor cameras (in red). The sensing area of each sensor is drawn by blue circles and the radio coverage area (two times the sensing coverage area) is drawn in yellow. This system is also called hexagonal grid. It allows covering all the sensing field while maintains each sensor connected with at least two or three neighbors. Bearing in mind that c is the radio coverage radius and s the sensing coverage radius, the distance between sensors to avoid holes without sensing field is $\sqrt{2}\cdot s$.

Now, we can estimate the overlapping sensing area (see A in Figure 4) taking into account the circular segment equation [39]. This is given by Equation (5):

(5) $$A=0.864\cdot s^{2}$$

The hexagonal grid is the worst among all since it has the biggest overlapping area [40], but it is the one that covers the entire sensing field.

The area covered by all the WSN as a function of the number of nodes *n* when n ≥ 2 in the WSN is expressed by Equation (6):

(6) $$A=n\cdot \pi \cdot s^{2}-0.864\cdot a_{n}$$

where $a_{n}=\sum_{i=1}^{n}\left(a_{n-1}+{\left(-1\right)}^{n}\right)$, n ≥ 2 and a_1_ = 1.

### 3.2. Wireless Sensor Node

In order to design our sensor node, several factors must be considered. On the one hand, the node must be able to capture the images with enough quality and the image processing must be computed inside the node in order to detect any unusual status of the leaves that could be caused by a deficiency, pest, disease or other harmful agents. When any symptom is observed in a leaf, the node must send an alarm to the gateway node.

We decided to use an available access point or wireless router on the market in order to reduce the costs of node deployment and take advantage of existing hardware. The main features of the needed hardware are that it must use IEEE 802.11a/b/g/n technology and it must also have at least one USB port in order to add a camera. The device should have the best hardware features because it must be able to process images. Moreover, the selected device for image capture, such as a video camera or webcam, must have USB and must adapt its hardware characteristics to the hardware characteristics of the access point or wireless router. Finally, the camera should be able to rotate, with the aim of having a larger viewing area and therefore reduce the number of required sensor nodes.

In general, the original firmware of the access points and the wireless routers on the market does not allow some operations. Therefore, we should customize the device firmware in order to enable these additional features.

One of the most used custom firmwares is OpenWRT [41]. It is a distribution based on GNU/Linux used for embedded devices such as routers and access points. It was driven initially by the GPL license and its intention was to allow manufacturers and users to modify and improve the code of their devices. OpenWRT provides a fully writable file system with package management. It is a very basic operating system that offers the possibility of complement it, with additional functionalities, using packages that allow users full customization ability to particularize the use of the device to a desired application.

There are many devices that can operate under the OpenWRT firmware. In order to design our system, we need a wireless device that has to be able to work on IEEE 802.11 standard, and must have at least one USB 2.0 port. The USB port will be needed to connect a camera and acquire high quality images from the vineyard. It is important to choose the best hardware features in order to facilitate the tasks of image processing. Table 1 only shows those devices that meet our requirements [42]. It displays information about the type of hardware of the devices, CPU Speed, RAM and flash memory, number of USB ports and the version, the type of wireless card used and the wireless technology used, among other features.

We selected the routers with higher processing capacity because we need to process images. The models with higher speed processors are the Netgear WNDR3700 and D-Link DIR-825. Both work with an Atheros AR7161 processor, (32 bit MIPS 24K processor core) [43], with a CPU Speed of 680 MHz and 64 MB of RAM memory.

Moreover, we should keep in mind that our system will be mounted on a field, where it is difficult to get energy from the electrical grid in many cases. In these situations, it is common to use batteries and electrical power generation systems from alternative sources. Therefore, another factor that was taken into account was the energy consumption of the device. Table 2 shows the energy consumption in active mode, idle mode and standby of some devices (we have included in this table only the ones for which some consumption information can be found in their technical specifications and the ones of whose consumption information is published in a research paper). Active mode occurs when the device is transmitting or receiving and processing, Idle mode occurs when the device is neither transmitting nor receiving or processing, but it is able to receive and process, and Sleep mode (or Standby mode) occurs when it is able to process or receive, but not to transmit. We have written a dash “–” when the information was not found. Some values have been provided using the mean values of the measurements taken during a period of time.

Focusing only on the ones with higher processing capacity (which is the purpose of our research), Table 3 shows a comparison in terms of weight and energy requirements. Even its size and/or its weight can be important for the node design.

Finally, we chose the Netgear WNDR3700 device. This router is capable of operating at frequencies of 2.4 GHz and 5 GHz, with transfer speeds up to 300 Mbps. It also incorporates mechanisms to implement QoS and security systems to ensure the wireless network integrity as a double firewall, DoS, IDS, WPA, WPA2, and WEP.

### 3.3. Camera

Another important element in the sensor node is the device for image capture. We analyzed different cameras and webcams available on the market. The first limitation was given by the hardware requirements forced on by the router or AP. Table 4 shows cameras that meet these requirements.

Among all of them, we chose the Hercules Webcam Classic, because it is the camera with better features. It is a small camera with a resolution of 1.3-Mpixel (1,290 × 960) and it can work with both USB 1.1 and USB 2.0. This resolution let us obtain different image sizes as a function of the desired quality of image. Table 5 shows the sizes that can be obtained, depending on its quality in dots per inch (dpi). A picture quality between 250 and 300 dpi is considered a good quality image. Chosen cameras obey that the sensing coverage area is approximately a half of the radio coverage radius.

### 3.4. Rotation System

Finally, we provided mobility to the node with the aim of increasing the viewing angle of the camera. It allows us to reduce the number of nodes required to monitor the area.

There are different ways of implementing this system. However, we aim to implement an economical and simple low energy cost option, but 100% functional. Our system is based on the control of camera movement, using a stepper motor, which is controlled by a PIC that has a small program inside. Stepper motors are commonly used for the construction of mechanisms in applications that require precise movements.

**Figure 5.**
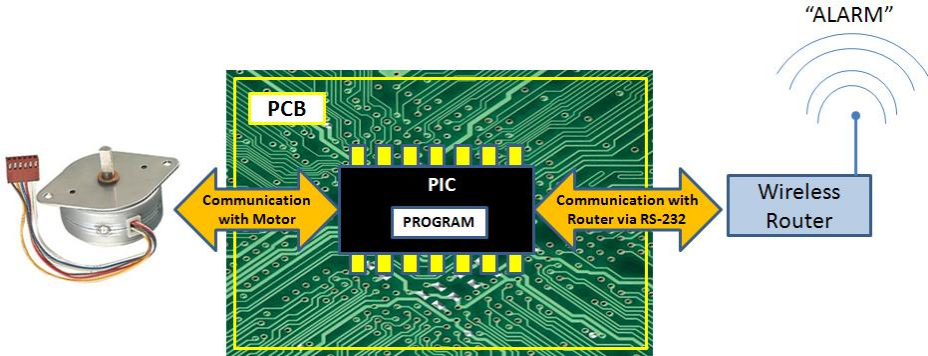
Stepper motor controller.

The system operation is very simple. The sensor node rotates on itself and it periodically captures images and processes them in order to determine if there is any unusual status of the leaves or not. The number of degrees, that the camera is able to rotate at every turn, can be as small as 0.72°, and up to 90°, depending on the selected motor. The number of times that the camera has moved (respect to its initial position) can be stored and sent when this information is required. As we can see in Figure 5, the information about the position of rotation is sent via RS-232 to the router, which is equipped with an RS-232 interface. The router is responsible for sending the alarm signal with this value.

**Figure 6.**
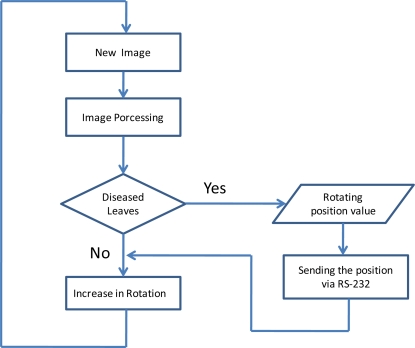
Program diagram.

When the node detects a symptom in the processed image, it sends the position value jointly with the alarm signal. Thus, knowing which node has generated the alarm and the position of the camera, it is possible determine exactly, what field area is sick. Figure 6 shows a simplified program diagram that would perform this operation.

In order to encode the position value, we must consider, first the type of motor that will be used. For our application we use a stepper motor, with rotation increments of 1.8°. This means that we could go through 360 degrees in 200 steps of 1.8°. Moreover, we note that signals that are sent through the serial port have a data field of 8 bits (without taking into account, start bit, stop bit, parity bit, *etc.*). Therefore, we can identify each motor step with an 8-bit binary value, being “00000000” 0° and “11000111” would be 358.2 degrees.

**Figure 7.**
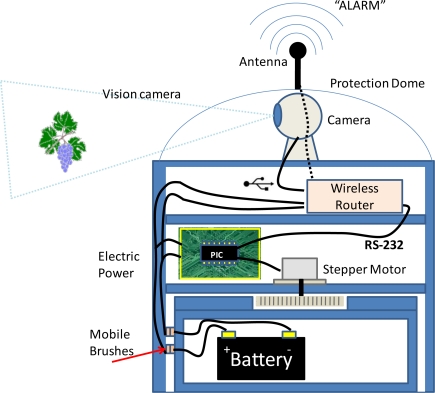
Sensor Node.

Moreover, if we analyze the mechanic part of the development, we can make the system capable of rotating 360 degrees, using a mobile brush for electric power transmission to the router and the engine. Figure 7 shows a cross section of the node once assembled. This is a shell composed of a fixed base that holds the battery and another moving part that protects the camera, router, and the stepper motor. The union between the two sides is made by epicyclical or planetary gearing and an annulus gearing, in order to transmit rotary motion from one place to another. The use of devices capable of performing turns of 360 degrees, reduces the number of devices to be installed along the area being monitored.

## 4. Bad Leaves

We define bad leaves those ones that show a visible unusual status that can be associated to a symptom of any deficiency, pest, disease or other harmful agent. There could be deficiencies, pests, diseases or other harmful agents that could not be easily detected from an image, so our system will not be able to detect it as a bad leaf and it will need more than a visual inspection in order to know the symptom.

A disease causes morphological or physiological alterations in the vine plant. The disease could be caused by fungus, germs, viruses or other pathogens. It can be detected because, in many cases, some color stains appear on the leaf. So, the symptoms can be visually detected because of the change of the green color. Some viruses are not easily detectable because their manifestations can only be detected by a mosaic in the leaf or by the leaf deformation. These last cases cannot be currently detected by our system, but we hope to find a way of including pattern detection in our system and make them detectable.

Pests are a set of insects that eat the vine plant. They eat or bite the leaves. They can be easily recognized because there are some bites or holes, or parts of the leaf are missing. The type of symptom depends on the insect. For example, the worm usually eats the leaf from the border to the center. Only those pests that cause a change of color in the leaf (because they live there or because the eggs cause an external color change) are detected.

When there are some chemical elements in excess or defect, a deficiency symptom appears. It could happen because of the need of some essential elements such as nitrogen, phosphorus, magnesium, potassium, calcium, and sulfur. But, the trace elements, that are needed in little quantities, such as iron, manganese, boron, copper, zinc and molybdenum, could also cause deficiencies. Some of them cause the color of the leaf to change to light green, yellow, brown, black, *etc.*, which could be detectable by our system, but other symptoms such as short size, different thickness, *etc.* will not be detectable by it.

Some symptoms would appear to be the same for different cases, so our system is only able to detect that it is a bad leaf that has a visible unusual status which is caused by any of these systems, thus an alarm should be sent to the farmer.

## 5. Image Processing

The objective of the image processing is to obtain a measure of the amount of bad leaves in the area where the vineyard is placed, which depending on this value would mean that the plant has a problem and need to be watched. The difficulty is that the bad leaves can be confused with the ground in many cases, because they can have similar colors, and then other processing is needed. Another problem is that the images are not taken from the same distance to the vines, and this implies that the size of the leaves is different from image to image. At the end of the process we will get a mask with all the pixels that are estimated to be part of the bad leaves.

The system has been deployed in Spain, where the mean value for the hours of sun varies from 1,584 to 3,433 depending on the region. Concretely the system is deployed to be used from 1 May to 30 September (when the vineyard plants grow, depending on the variety of the grapes). In Spain, there is usually an outdoor environment with good illumination conditions. Spring and summer seasons have a mean value between 11 and 12 hours per day, which provide good clarity to ensure an accurate image processing and obtain good decisions. Although we have not included illumination compensations in order to correct the brightness changes, the system is developed with the objective of adding new features. In future works we will add chromaticity-based approaches [49,50] in order to correct the brightness changes due, for example, to the presence of clouds or rain.

**Figure 8.**
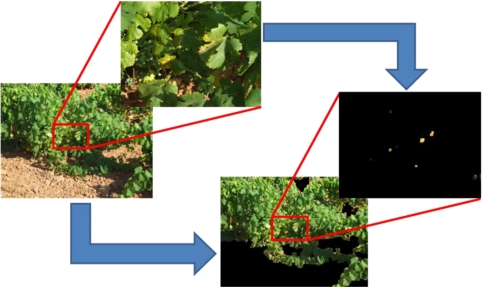
Detection of brown leaves in vines.

We show an example of the detection problem in Figure 8. When the plants are distant, probably we won’t have enough resolution to accomplish with the entire processing, but we can use image processing to detect where the leaves are. In a further step, by using the camera zoom that enables to get images with a higher resolution, we could be able to detect the bad leaves, although they must be first distinguished from the ground.

**Figure 9.**
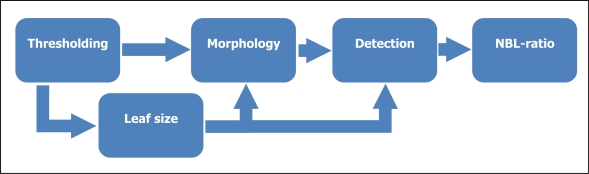
General schema followed in the image processing stage.

Figure 9 shows the process followed. It has been separated into five stages. In the first, two conditions based on the color of the pixels are applied to the images. The first one is used to detect where the good leaves are, and will be used also to estimate an average leaf size that will be used in later stages. Then, a threshold is applied to the remaining pixels in order to discard those that don’t match a color condition corresponding with the bad leaves. After this stage, we get a detection mask composed of groups of pixels which their color is similar to the bad leaves, but there will be some groups that are not interesting because they don’t correspond to what we want to detect.

The next stage is a set of operations in order to reduce the noise in the detection mask that happens when a group is too small or there are two groups too close. An important issue to take into account for these operations is the leaf size in the image, because when it is small, the operations should not delete small but significant groups, and when it’s large there is a bigger margin to smooth the mask.

In the detection stage, another threshold, based on the leaf size too, is imposed to each group of pixels to differentiate between the ground and the interesting part. Groups that are higher than this threshold are discarded, and the others make up the final detection mask.

Finally, a parameter, that is called *number of bad leaves* (NBL), is calculated as the number of pixels in this final mask relative to the leaf size. Then, this parameter is used by the node in order to take a decision about the level of bad leaves on the image and to generate the alarm in the corresponding case. This is done by means of a threshold that could be fixed experimentally attending to each particular case.

The generation of this alarm can also be taken considering several images and their different values of NBL, although, in this case, others factors should be taken into account, like the uncertainty about every result related to the resolution of the image. In this sense, data fusion techniques could be attempted in order to take the decision. In the following, each stage is explained in more details with examples.

### 5.1. Thresholding

The objective of this stage is to get a first mask of detections with all the pixels that have a similar color to what we want to detect. This goal is accomplished with two conditions based on the color of the pixels, assuming that good leaves have a green color and bad leaves have a color similar to brown, but covering many more color shades.

**Figure 10.**
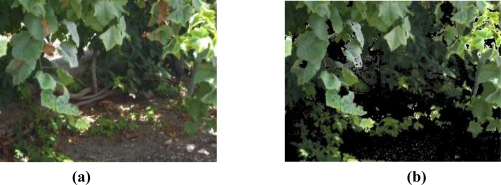
**(a)** Example of a typical image to be processed. **(b)** Pixels detected as good leaves, that is, applying the green condition.

A first condition detects good leaves in order to discard them. Therefore, it is compound of all the pixels which highest RGB component is the green. Figure 10(a) shows an example image, where there are several bad leaves, both in the tree and on the ground, which are the target to be detected. Also, is important to note that the ground can be easily confused with the bad leaves. The good leaves are easily detected with this “green condition”, as shown in Figure 10(b).

**Figure 11.**
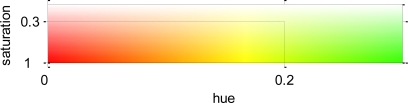
Colors included imposing the thresholds to HSV values (saturation is 1).

The second condition is applied on the remaining pixels after discarding the previously detected. It uses a previous transformation of the original image from RGB values to HSV (Hue Saturation Value). This is a non-linear transformation that changes the RGB values in a way that corresponds more naturally to human perception. This allows setting thresholds to hue and saturation values of the pixels in order to detect the target colors. More precisely, a maximum threshold on the hue component have been fixed to TH = 0.2, and a minimum threshold of TS = 0.3 for the saturation, which have been adjusted to be appropriate for the simulations done. Figure 11 shows the colors included by these thresholds, which covers a great variety of hues.

**Figure 12.**
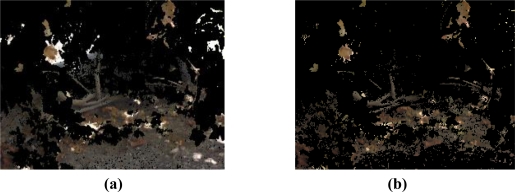
**(a)** Pixels remaining after applying the green condition. **(b)** After applying the second condition.

Figure 12(b) shows the result of applying this second condition to the example in Figure 10. In Figure 12(a) it is shown the remaining pixels of the example image after discarding the pixels with the green condition, which will be the pixels on which this condition will be applied. We can see how, besides the green pixels, others pixels of the ground and the back of the image have been discarded, keeping the pixels of the bad leaves. However, the image has a lot of noise that should be removed.

### 5.2. Leaf Size

Based on the pixels that compound the good leaves, which were obtained by applying the green condition explained in the previous stage (see Figure 10(b)), we have implemented an automatic estimation of an average leaf size in the image, since this result will be required in later stages. The algorithm is based on the idea that, as there are more leaves in the image, the different orientations and shapes leads to a higher variability of the intensity of neighbor pixels in the image.

First, the image is divided in non-overlapping squared blocks of pixels, and only blocks that all their pixels accomplish with the green condition are used. To reduce different lighting conditions of each image, a normalized difference index (NDI) that uses green and red components of the RGB values of the pixels is used [51]. NDI follows Equation (7):

(7) $$NDI=\frac{green-red}{green+red}$$

For each block, the mean of this value for all its pixels is calculated, and then the difference of this value with the mean of adjacent blocks. Finally, the global “variability” of the image is given by the mean of these differences.

**Figure 13.**
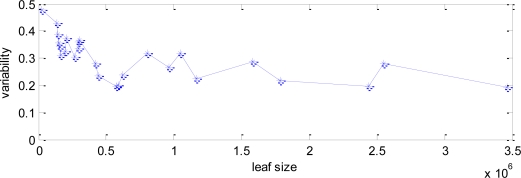
Variability of several images in function of the leaf size.

To test the validity of this method, several images have been processed, which have different leaf sizes since they are taken from different distances to the trees. An estimation of an average leaf size has been manually made for each one, and these values have been compared with the distance obtained after processing the images. Figure 13 shows this comparison, with the values of the manually-assigned leaf size in the x-axis, and the difference value in the y-axis.

There is a decreasing trend of the difference value as the leaf area increases, which confirms that this difference is higher for small sizes. However, there is a great deviation for any particular image. There are several factors that can affect this relation, such as the lighting conditions, as said. At the same time, the leaf area assigned to each image is done manually, and so it is subjective to the operator. Also, we have assumed that one image have the same size for all its leaves, but this is not always true, since depending on the perspective, there can be leaves of very different sizes.

Taking into consideration these results, we have defined roughly three range of variability to be assigned to three leaf sizes, small, medium and large. The parameters that have to be adjusted depending on this value will be set to the same value for each range.

### 5.3. Morphology

The objective of this stage is to give some consistency to the mask obtained previously. Taking advantage of the fact that the pixels detected are grouped together, we can give more significance to some of them considering their number of pixels and shape. There are two main goals: to delete those that are too small to be a relevant detection, and to merge together several groups that are close together. The two steps are applied consecutively, using *morphological operators* [52] on the image, which are a set of techniques that process images based on shapes. They work applying a structuring element that determines the size and how the operation is done, and output an image of the same size. There are two basic operators, that are *erosion* and *dilation*, which adds or removes pixels to the boundaries of objects, respectively.

**Figure 14.**
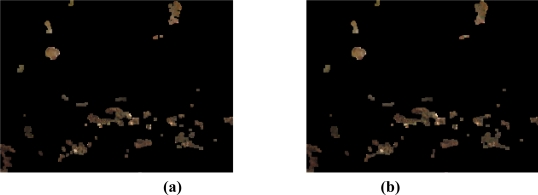
Image of Figure 12(b) after morphological operations with a squared structuring. **(a)** After the closing operation. **(b)** After the opening operation.

The first goal is accomplished with a *morphological opening operation* on the mask, and consist in an erosion of the image followed by the dilation of the eroded image. An example of this operation on the image of Figure 12(b) can be seen in Figure 14(a). Following, there is a *morphological closing*, which is the inverse operation, and that achieves the second goal, as can be seen in Figure 14(b). The resulting mask has smoothed boundaries, holes filled, and small groups of pixels removed.

The size of the structuring element must be related with the previously calculated leaf size, and must be small enough to not remove significant detections in the opening step, but also must be big enough to fill holes and join detections that are close together.

### 5.4. Detection

The objective of this stage is to classify the different detections into good ones and bad ones. Mainly, the purpose is to differentiate between detections that are brown leaves and those that are ground. For that, a condition of maximum size of the detection is imposed. This value must be also relative to the distance from which the image is taken, and therefore have been fixed relative to the leaf size previously obtained.

After this condition is applied a mask with only the significant detections is obtained. A ratio of the *number of bad leaves* (NBL) in the image is then calculated as it is shown in Equation (8):

(8) $$NBL=\frac{\%\;\text{pixels in the mask}}{\text{size of leaf}}$$

This value takes into account both the size of the detection mask and the size of the leaf, and gives an idea of how bad the plants in the image are.

An important question to take into account is the image resolution needed in order to get good results. We expect that as the resolution of the vines is lower, the results will be worse. For this purpose a study have been done processing a set of images, for which the NBL ratio have been obtained. Also, the good detections in this images have been manually marked in order to get a truth-mask to compare the results.

**Figure 15.**
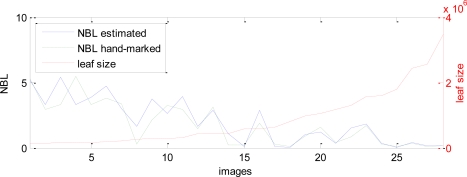
Number of brown leaves (NBL), for different images, estimated with our algorithm (solid line) and hand-marked (dashed line) for comparison. Images are sorted by the leaf size, also showed (dash-dotted line).

Images have been taken from different distances and with different perspectives, including or not including ground, sky, … For each one, an average leaf size in pixels have been manually assigned, and since all the images have the same size, this is directly related with their resolution. In Figure 15, the results for the different images are shown, showing both NBL, the estimated and the obtained from hand-marked mask. They are sorted by the leaf size (showed as dash-dotted line), that is, by increasing resolution.

**Figure 16.**
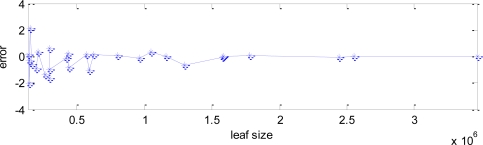
Error in the estimated NBL compared with the leaf size.

The error in the estimation of the NBL compared with the hand-marked mask, calculated as the difference in NBL obtained in both cases, is shown in Figure 16, as a function of the leaf size. The error is clearly decreasing as the size raises, that is, as the resolution is higher because the images are taken from a closer distance.

This last result means that this technique is only useful with a certain resolution of the plants. However, if the resolution is not enough, the cameras can make a zoom at the vines (detected by the green mask) and perform a better processing.

**Figure 17.**
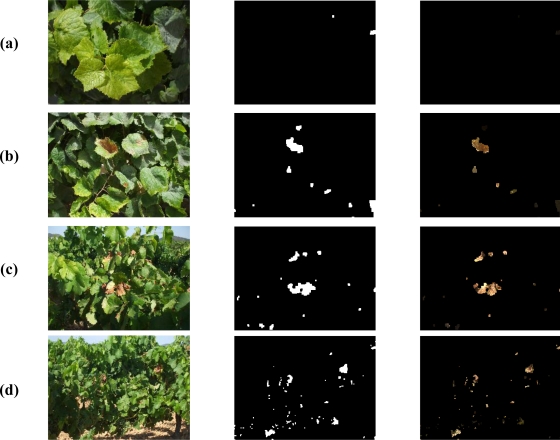
Four examples of detections in images taken from different distances. In each case, we show the original image in the first column, the final detection mask obtained in the second, and the masked image in the third. **(a)** An example of an image with large leaves. **(b)** and **(c)** Examples of medium leaf size. **(d)** Example of small leaf size.

Finally, we show several examples of the processing in Figure 17. There are involved images taken from different distances, and therefore different leaf sizes.

In Figure 17(a) there is an example of an image where all the leaves are good. The leaves are large since the image has been taken from a short distance with a good resolution. We can see two detections that should not be detected, but they are very small compared to the leaf size, leading to a small value of NBL. The NBL for this image is 0.02.

Figure 17(b,c) shows examples of images with medium leaf size, taken from a distance higher than the previous case. The resolution is medium also. Bad leaves are correctly detected and at the same time we can see that the sky in the second example is discarded. Also, there are small detections that could be interpreted as erroneous, but it seems that the second image have a more quantity of leaves with problems than the first one. This is confirmed by the NBL estimated, that is 0.85 and 1.42, respectively.

Finally, in Figure 17(d), there is an example of an image taken from a far distance, and therefore we have a low resolution. As can be appreciated, the leaves are small compared with previous examples. Although the sky have been correctly discarded and also most of the ground, we can see a higher number of erroneous detections in this part of the image, some of them have a significant area. For images with this low resolution, the precision of the results are not so good as before. The NBL in this case is 2.5.

## 6. Sensor Network Traffic Measurement

**Figure 18.**
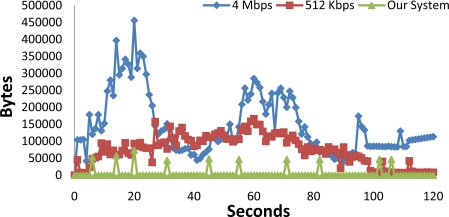
Bytes comparison when there is 4 Mbps video stream, 512 kbps video stream and our system.

In order to show the benefits of the proposed system, in this section we compare the traffic measurement provided by a single sensor node that takes images of a 360° of view from the vineyard field and transmits them to a central server, in order to analyze the images, with our sensor node that only sends one image (when it founds a bad leaf while going around 360°). In order to have enough image quality, the images had a resolution of 640 × 480 pixels and a frame rate of 30 fps. No audio was transmitted. Figure 18 shows a comparison of two video bit rates (4 Mbps and 512 kbps) with our system, which sent a picture of 640 × 480 pixels. The camera needed 120 seconds to make a round of 360°. We can see that there are more bytes sent to the network for the 4 Mbps video stream. There is a mean value of 146,130.64 bytes per second. When the 512 kbps is sent there is a mean value of 74,785.76 bytes per second. In our case, the picture was sent in the 20th second. There are other packets in the network because of the regular procedure of the WSN. In our case there is a mean value of 4,259.86 bits per second. Now we can see that, in terms of bytes transmitted, there is a decrease of 97.09% from the 4 Mpbs to our system and a decrease of 94.30% from the 512 kbps to our system. Bearing in mind that the most power consumption issue in a sensor to transmit bytes through the wireless interface [53], we can state that our system saves energy.

**Figure 19.**
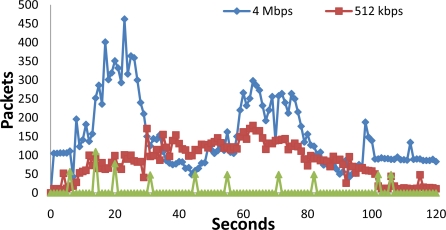
Packets comparison when there is 4 Mbps video stream, 512 kbps video stream and our system.

Now, in Figure 19, we compare the number of packets sent to the network for the 4 Mbps and the 512 kbps video streams with our system. We can see that there are quite less packets sent to the wireless in our system. Only the forwarded messages and the image sent in the 20th second can be seen in the network. 4 Mbps video stream is the one that sends more packets.

These measurements show that our system saves energy because there are less packets and less bytes transmitted to the wireless medium.

## 7. Conclusions

In this paper we have shown a WSN that uses an image processing system in each wireless node to detect any unusual status of the leaves that could be caused by a deficiency, pest, disease or other harmful agent in the vineyard. When the wireless sensor detects any symptom in the vine leaves, it generates an alarm that is routed to the sink node in order to warn the farmer. We have studied the WSN in terms of sensing coverage area and radio coverage area. The distributed design allows lower bandwidth consumption, and higher scalability than when all video streams are transmitted through the network, as we have shown with the sensor network traffic.

In order to detect bad leaves we have designed a 5-stage process. The first one is used to detect where the good leaves are and it also estimates an average leaf size that will be used in later stages. Then, a threshold is applied to the remaining pixels in order to discard those that don’t meet a color condition corresponding with the bad leaves. The next stage is a set of operations in order to reduce the noise in the detection mask that happens when a group is too small or there are two groups too close. In the detection stage, the leaf size is used as a threshold in order to differentiate between the ground and bad leaf. Finally, NBL parameter is calculated as the number of pixels in this final mask relative to the leaf size. Then, this parameter is by the node to take a decision about the level of bad leaves on the image and to generate the alarm in the corresponding case.

Taking into account the information gathered in the sensor network traffic measurement section about the mean value of the traffic (4.26 kbps), and bearing in mind that in a IEEE 802.11g has a theoretical bandwidth rate of 54 Mbps, but an effective bandwidth rate of 27 Mbps, we can state that there will not be any limitation on the number of nodes operating in the WSN at least from a theoretical point of view.

In future works we will add chromaticity-based approaches in order to include illumination compensations and correct the brightness changes due, for example, to the presence of clouds or rain.

We think that by combining thresholding strategies with artificial neural networks for image processing, and learning from the experience, we will be able to obtain more accurate results. Moreover, we are also thinking about including an image recognition database and a neural network in order to distinguish between deficiencies, pests, diseases or other harmful agents, and our future works are focused on this research line.

The next step in our research is to include energy saving methods and collaborative group-based systems (developed by some of the authors of this paper) in order to enhance the system and obtain higher performance with lower energy consumption [54,55].

## Figures and Tables

**Table 1.** Available hardware comparison for sensor nodes.

**Brand**	**Model**	**Version**	**Status**	**Target(s)**	**Platform**	**CPU Speed (MHz)**	**Flash (MB)**	**RAM (MB)**	**Wireless NIC**	**Wireless Standard**	**Wired Ports**	**USB**

Alcatel-sbell	RG100A-AA	Rev 0	10.03	brcm63xx	Broadcom 6358	300	16	32	BCM4318	11a/b/g	4	1
Asus	WL-500g Deluxe	?	8.09	brcm-2.4 brcm47xx	Broadcom 5365	200	4	32	BCM4306 r5	11b/g	5	2× 2.0
Asus	WL-500g Premium	v1	8.09	brcm-2.4 brcm47xx	Broadcom 4704	264	8	32	BCM4318	11b/g	5	2× 2.0
Asus	WL-500g Premium	v2	8.09	brcm-2.4 brcm47xx	Broadcom 5354	240	8	32	BCM5354	11b/g	5	2× 2.0
Asus	WL-520gU	?	8.09	brcm-2.4 brcm47xx	Broadcom 5354	200	4	16	Broadcom	11b/g	5	1× 1.1
Asus	WL-HDD2.5	?	10.03	brcm-2.4 brcm47xx	Broadcom 4710	125	4	16	BCM4706	11b/g	1	1× 1.1
Asus	WL-600g	?	10.03 no adsl	brcm-2.4 brcm63xx	Broadcom 6348GW	256	4	16	BCM4318	11b/g	4	2× 2.0
Buffalo	WZR-HP-G300NH	v1	10.03	ar71xx	Atheros AR9132	400	32	64	AR9103	11b/g/n	5 gigE	Yes
D-Link	DIR-320	A1	10.03	brcm-2.4 brcm47xx	Broadcom BCM5354	240	4	32	Broadcom 4318	802.11b/g	5	Yes
D-Link	DIR-825	B1, B2	10.03	ar71xx	Atheros AR7161	680	8	64	Atheros AR922x	802. 11a/b/g/n	5 gigE	Yes
Fon	Fonera2	FON2202	10.03	atheros	Atheros AR2315	180	8	32	Atheros	11b/g	2	1× 2.0
Linksys	WRT160NL	1.0	10.03	ar71xx	Atheros AR9130	400	8	32	Atheros AR9100	11b/g/n	5	Yes
Linksys	WRT350N v2	2.0, 2.1	10.03	orion	Marvell 88F5181L	500	8	32	Atheros AR5416	11b/g/n	5	Yes
Linksys	WRTSL54GS	1.0, 1.1	0.9	brcm-2.4 brcm47xx	Broadcom 4706	264	8	32	Broadcom	11b/g	5	1× 2.0
Linksys	WRT54G3GV2(-VF)	1.0	10.03	brcm47xx	Broadcom 5350	264	16	32	Broadcom	11b/g	5	3× 2.0
Netgear	WNDR3700	?	10.03	AR71xx	Atheros AR7161	680	8	64	Atheros AR9280 bgn /Atheros AR9280 an	11a/b/g/n	5 gigE	1× v2.0
Netgear	DG834GT	?	10.03	brcm63xx	Broadcom 6348	255	4	16	Atheros	11b/g	4 + ADSL	1× v1.1
Planex	MZK-W04NU	?	10.03	ar71xx	Atheros AR9130	400	8	32	Atheros AR9100	11b/g/n	5	1× 2.0
TP-Link	TL-MR3420	1	trunk	ar71xx	Atheros AR7241	400	4	32	Atheros AR92xx	11b/g/n	5	1× 2.0
TP-Link	TL-WR741ND	1	10.03	ar71xx	Atheros AR7240	400	4	32	Atheros AR92xx	11b/g/n	5	Possible 1× 1.1
TP-Link	TL-WR741ND	1.9	trunk r23058	ar71xx	Atheros AR7240	400	4	32	Atheros AR92xx	11b/g/n	5	Possible 1× 1.1
TP-Link	TL-WR941ND	2	10.03	ar71xx	Atheros AR9132	400	4	32	Atheros AR9100	11b/g/n	5	1× Header
TP-Link	TL-WR941ND	3	10.03.1	ar71xx	Atheros AR9132	400	4	32	Atheros AR9100	11b/g/n	5	1× Header
TP-Link	TL-WR941ND	4	10.03	ar71xx	Atheros AR7240	400	4	32	Atheros AR92xx	11b/g/n	5	1× Header
TP-Link	TL-WR1043ND	1–1.6	10.03	ar71xx	Atheros AR9132	400	8	32	Atheros AR9100	11b/g/n	5 gigE	1× 2.0

**Table 2.** Energy consumption comparison.

**Brand**	**Model**	**Active Mode (Watts)**	**Idle Mode (Watts)**	**Sleep mode or Standby mode (Watts)**

Alcatel-sbell	RG100A-AA	7	5	3
Asus	WL-HDD2.5	4.56	3.12	2.38
Buffalo	WZR-HP-G300NH	5	–	–
D-Link	DIR-825	7	–	–
Fon	Fonera2 FON2202	4.8	4.15	2.15
Netgear	WNDR3700	6	4.77	2.57
Netgear	DG834GT	5.19	4.92	4
TP-Link	TL-MR3420	8	–	–
TP-Link	TL-WR741ND	4	–	–
TP-Link	TL-WR1043ND	9	6.9	–

**Table 3.** Weight and energy requirements comparison.

**Brand**	**Model**	**Power Requirements**	**Weight**

Netgear	WNDR3700	12 V/1 A	500 g
D-Link	DIR-825	12 V/2 A	900 g

**Table 4.** Camera comparison.

**Camera Model**	**Sensor**	**Resolution (pixels)**	**System Requeriments**	**USB Version**

Hercules Classic Webcam [44]	VGA	1,280 × 960	CPU Processor 500 MHz, 64 MB RAM Memory.	USB 1.1 and USB 2.0
ClickSmart 420 [45]	CMOS VGA	640 × 480 (video) 1,280 × 960 (photography)	CPU Processor 500 MHz, 64 MB RAM Memory.	USB 1.1 and USB 2.0
QuickCam Cordless [45]	CMOS	510 × 492	CPU Processor 400 MHz, 64 MB RAM Memory.	USB 1.1 and USB 2.0
QuickCam for Notebooks Pro [45]	CCD VGA	640 × 480	CPU Processor 400 MHz, 64 MB RAM Memory.	USB 1.1 and USB 2.0
Creative WebCam NX Pro [46]	CMOS VGA	1,024 × 768	CPU Processor 233 MHz, 32 MB RAM Memory.	USB 1.1 and USB 2.0
Creative WebCam Instant [47]	CMOS VGA	352 × 288 (video) 640 × 480 (photography)	CPU Processor 266 MHz, 64 MB RAM Memory.	USB 1.1
A4tech PKS-635K [48]	CMOS VGA	640 × 480	CPU Processor 166 MHz, 32 MB RAM Memory.	USB 1.1 and USB 2.0

**Table 5.** Image size comparison.

**Size (pixels)**	**80 dpi (average monitor quality)**	**133 dpi (in cm)**	**155 dpi (in cm)**	**175 dpi (in cm)**	**200 dpi (in cm)**	**250 dpi (in cm)**	**300dpi (in cm)**

1280 × 960	30.4 × 40.6	18.3 × 24.4	16.3 × 21.7	13.9 × 18.6	12.2 × 16.3	9.8 × 13.0	8.1 × 10.8
